# The Effects of a Physically Active Lifestyle on the Health of Former Professional Football Players

**DOI:** 10.3390/sports7040075

**Published:** 2019-03-28

**Authors:** Tuba Melekoğlu, Erdi Sezgin, Ali Işın, Ayşen Türk

**Affiliations:** 1 Department of Trainer Education, Akdeniz University Faculty of Sports Sciences, Antalya 07058, Turkey; sezginerdi@hotmail.com (E.S.); isin_ali@hotmail.com (A.I.); 2 Clinics of Sports Medicine, Antalya Education & Research Hospital, Antalya 07058, Turkey; draysenturk@gmail.com

**Keywords:** former football players, exercise, health, weight gain, lifestyle, physical activity

## Abstract

The purpose of this investigation was to determine if a physically active lifestyle affects the health of former football players. Sixty former professional football players aged 40–50 years and who ended their sports career at least ten years ago were recruited for the study and grouped into two groups based on their physical activity habits after their retirement. Health and lifestyle characteristics were collected through a questionnaire to obtain information about recreational physical activity levels, diseases, family medical history, smoking, alcohol intake and dietary habits. Furthermore, lung functions, blood parameters and cardiovascular health were evaluated. Our results showed that body weight and body fat percentage were significantly higher in retired footballers who had a sedentary lifestyle compared to those who were physically active. The absolute and predicted values for forced expiratory volume in one-second values were higher in the active group. Twelve retired athletes were found to have intraventricular conduction delay. The findings suggest that former footballers who have higher levels of physical activity have advanced body composition, respiratory functions and serum lipids compared to former footballers with less active lifestyles. It is recommended that former elite athletes should maintain physically active lifestyles to sustain their health and reduce the risk of disease and disability in the later years of life.

## 1. Introduction

Football is the most popular branch of sport played throughout the world and is a physical game that requires high performance. The modern understanding of football requires players to be more flexible and versatile in terms of the qualities required by performance and game fields. Therefore, in order to play football professionally at a high level, athletes need to train intensively and extremely hard so that they can improve important physical attributes [1]. This training approach causes football players to experience several physical and physiological adaptations. These adaptations depend on extreme training and also influence the health parameters of athletes [1,2,3]. Moderate level physical activities are known to have positive effects on health, such as decreasing the risk of diseases of the cardiovascular system, musculoskeletal conditions and type 2 diabetes mellitus [4,5,6]. However, there is not much research about the effects of extreme physical activities on elite athletes. Furthermore, it is difficult to eliminate life habits and genetic factors affecting health, such as diet, smoking and levels of recreative physical activities, as confounding factors and thus, it is difficult to interpret the results of studies to evaluate the effects of high intensity exercises and related future risks of disease. 

There is strong evidence that regular physical activities play a protective role against some cardiovascular diseases, hypertension, stroke, metabolic syndrome, type 2 diabetes mellitus, breast and colon cancer and depression [4,5,6]. Some studies [7,8,9] have stated that extreme training might be harmful to the heart and might cause cardiac fibrosis and ventricular arrhythmia. Moreover, there is some evidence that lifelong extreme exercise decreases the risk of death and enhances functional capacities [7,10,11]. Some studies [12,13] have stated that participating in athletic activities that require standing for an extended period of time may play a role in the development of osteoarthritis. For example, some studies [14,15] investigating the musculoskeletal system have shown that former elite football players are 3–5 times more likely to get gonarthrosis compared to sedentary individuals and participating in extreme football training increases these risks. Besides, it is known that bone mineral density increases as the individual takes part in sports and this adaptation is considered to be permanent [16,17]. In some studies, it is pointed out that in former football players, their bone mineral density is higher and fracture risk is lower compared to sedentary individuals [16,17,18] but the study of Karlsson et al. [19] showed that this advantage disappears for those who are over 60 and 35 years after retirement and they subsequently have fracture risks similar to those in sedentary ones. 

Elite athletes terminate their sports career for various different reasons. If the athlete is retired due to injury, aging, deselection from a team or performance loss, a more difficult transition can be experienced [20,21]. Besides, it has been reported that the main reason for retirement is mostly sports injuries [22]. When someone unwillingly quits sports due to compulsory reasons, such as injuries, this might be followed by some negative psychosocial effects. Gouttebarge et al. [20] reported that the prevalence of anxiety and depression ranged from 16% to 39% in 104 former professional football players. As well as psychosocial effects, some of their life habits that continue after ending their active sports career also affect their health. In a study, former athletes who were physically inactive following sports career showed a low-coronary-risk lipid profile. Additionally, Pihl and Jürimae [1] revealed that weight gain during retirement was associated with a higher prevalence of cardiovascular disease risk factors in former athletes. Further, it was reported that physically active former athletes showed a significantly lower biological age compared to inactive former athletes [2]. In another study on former athletes [10], it has been reported that the levels of daily physical activities have more significant effects than being a competitor previously in terms of providing protection against illnesses. Although the risk of early death that is related to diabetes, hypertension, ischemic heart diseases and so on has decreased in elite athletes, it has been reported that continuing a physically active lifestyle has a more important contribution to health [10,23,24]. 

Although athletes participate in high levels of physical activities during their sports career, they may not transfer this into regular physical activity after retirement. The aim of this study is to compare some health parameters, such as body composition, blood markers and cardiopulmonary functions of former football players, according to their recreational physical activity level. We hypothesize that a physically active lifestyle has beneficial effects on some health indicators, such as body composition, serum lipid profiles, blood counts, respiratory functions and electrocardiographic results, in former football players.

## 2. Materials and Methods

### 2.1. Participants

Volunteers between 40–50 years old who played football professionally and ended their football career at least 10 years ago participated in the study. Former football players were grouped into two based on their physical activity scores according to the International Physical Activity Scale (IPAQ). Those who are very active and scored 3000 metabolic equivalent of task (MET)-minutes/week or above were grouped as the physically active group (AG, n: 30) and those who scored below 3000 MET-minutes/week were grouped as the sedentary group (SG, n: 30) [25]. Former football players at similar ages who took part in similar training and competitions were matched. 

Health and lifestyle characteristics were collected through a questionnaire to obtain information about diseases, family medical history, smoking, alcohol intake and dietary habits by a self-report method [26]. Family history and early death due to any illnesses were considered to be significant. For hypertension, the systolic blood pressure needed to be >140 mmHg or the diastolic blood pressure was >90 mmHg [27]. In order to assess the health of the participants, some tests were carried out, such as lung function tests, urinary tests, blood tests and electrocardiogram. 

All participants were informed about study procedures and signed a written informed consent form prior to the study commencement. The study was conducted in accordance with the Declaration of Helsinki and approved by the Akdeniz University Clinical Research Ethical Board (70904504/197; 09/09/2015).

### 2.2. Physical Activity Questionnaire (IPAQ) 

In order to measure the level of physical activity of the volunteers, the short form of the International Physical Activity Questionnaire (IPAQ) was used [25,28]. It is recommended that this questionnaire should be used on adults who are 18–69 years old. The questionnaire includes questions about minimum 10-minute physical activities within the last 7 days and it aims to determine how many days within the last week and how long (a) intensive physical activities, (b) moderate intensity physical activities and (c) walking were performed. The last question tries to assess how long a person sits in a day. The total physical activity score (MET-min/week) is calculated by converting the time periods of intensive and moderate activity and walking into MET, which accounts for basic metabolic speed.

### 2.3. Body Mass Index (BMI)

Height was determined without shoes on a portable stadiometer (Harpenden Portable Stadiometer, Holtain, Crymych UK) and body mass index (BMI) was calculated as weight in kg over height in meter squared. Based on WHO criteria [29], underweight was identified as BMI  <  18.5 kg/m^2^, overweight as BMI 25.0–29.9 kg/m^2^ and obese as BMI  ≥  30.0 kg/m^2^.

### 2.4. Bioelectric Impedance Measurements 

The body weight and body fat percentage of the volunteers’ bioelectric impedance measurements (BIA) were taken by Tanita Body Composition Analyzer Type SC-330 (Tanita, Tokyo, Japan). The measurements were taken while participants were motionless with both feet balanced on the scale. Participants were told to avoid intensive exercise and alcohol consumption 24 hours prior to testing and to avoid caffeine consumption at least four hours prior and food intake two hours prior to the testing [30].

### 2.5. Respiratory Tests 

In order to evaluate the respiratory health of the participants, their respiratory volumes and capacities were measured with Spirometry (Pony FX, COSMED, Italy). The spirometry measurements included forced vital capacity (FVC), forced expiratory volume in the first second (FEV1), peak expiratory flow (PEF), forced expiratory flow at 25–75% (FEF) and maximal voluntary ventilation (MVV) in 12 seconds. All spirometry tests were conducted according to American Thoracic Society (ATS) statement on the standardization of spirometry. The European Respiratory Society’s reference values (ERS 93) for spirometry parameters [31] were used to determine the percentage of predicted values of FVC (FVCp) and FEV1 (FEV1p).

### 2.6. Blood Tests

Blood samples were drawn in the morning after overnight fasting. All samples were analyzed in the same standardized laboratory. The blood samples were taken from the antecubital vein of participants for the analyses of the following: complete blood count, serum lipids and thyroid functions. Hemoglobin (Hb), hematocrit (Hct), red blood cell count (RBC) and white blood cell count (WBC) values in complete blood count parameters were evaluated. Triglyceride (Tg), low-density lipoprotein cholesterol (LDLc), high-density lipoprotein cholesterol (HDLc) and total cholesterol (Tc) were measured as serum lipids. The assessment of the thyroid function included measurements of serum concentrations of thyroid stimulating hormone (TSH), free thyroxine (FT_4_) and free triiodothyronine (FT_3_). 

### 2.7. Electrocardiography (ECG):

The ECG of the volunteers was recorded as 25 mm/s with a 1 L frequency ECG in the supine position after resting for 5 min. In the ECG results, the rhythm, heartbeat pace, QRS and T axes, QRS-T angle, PR, QRS, QT periods, the amplitude and period of P wave, QRS amplitude, R/S rate, the existence of pathological Q wave, ST segment and T wave were all evaluated in detail by an expert cardiologist by using descriptive categories from Pelliccia et al. [32]. For sinus bradycardia, the resting heartbeat was defined as a heart rate below 60 bpm.

### 2.8. Analysis of the Data

All values are reported as means ± standard deviations (SD). The normality and homogeneity of variance assumptions for all data were verified using Shapiro–Wilk and Levene’s tests. The assumptions of the normality and homogeneity of the data in the research were tested using the results of Shapiro–Wilk, Levene tests and the values of skewness–kurtosis. Parametric tests were applied to the groups with normal distribution and non-parametric tests were applied to the data not meeting these conditions. In order to evaluate the difference between the groups, the Independent Sample T-Test (parametric) and Mann–Whitney U Test (non-parametric) were applied. Statistical analyses were performed using IBM SPSS Statistics for Macintosh, version 23.0 (IBM Corp., Armonk, NY, USA). The analysis of the effect sizes (ES) was performed using G-power software (University of Kiel, Kiel, Germany), version 3.1. Effect sizes are expressed as Cohen’s d, with d  =  0.2 considered to be a small effect size, d  =  0.5, medium and d  =  0.8, large. The statistical significance level was set at p < 0.05 [33].

## 3. Results

No statistically significant differences in alcohol consumption, dietary habits and frequency of smoking were found between groups. Physical variables, including body weight, body mass index and body fat percentage, were found to be significantly higher with the large effect size in former football players who had a sedentary lifestyle compared to those who were physically active (p < 0.001). According to WHO’s criteria, the mean values of BMI were in overweight ranges for both groups. However, SG has significantly higher BMI results than AG. The physical variables of the participants are shown in Table 1.

FEV1 and FEV1p values were found to be significantly higher in AG than SG (p < 0.05). No statistically significant differences were found in other spirometry parameters (p > 0.05). Furthermore, SG was found to be below expected reference values for FEV1 and FVC averages. The spirometry parameters are shown in Table 2.

There were no statistically significant differences in blood parameters between groups. Although no statistically significant differences were found, it can be seen that Tg, Tc and LDL values of SG were higher than AG. The blood counts and thyroid function parameters were found to be within the normal reference ranges. The blood parameters of the participants are shown in Table 3.

A similar number of sinus bradycardia cases was diagnosed in the ECG of participants from both groups (8 and 10 for AG and SG, respectively). The resting heartbeat is similar in both groups (62.87 ± 9.16 and 66.43 ± 11.58 for AG and SG, respectively; p = 0.653). However, nine people in SG and three people in AG were seen to have intraventricular conduction delay and one person in both groups was found to have left anterior fascicular block.

## 4. Discussion

It is known that participating in regular physical activities provides positive contributions to health and longevity [34]. It has been reported that the death risk decreases by 20–35% as the level of sports participation increases [34,35,36]. Furthermore, some studies have shown that the risk of death caused especially by cardiovascular diseases decreases by up to 50% if the person is fit or active [5]. However, some studies [37,38] pointed out that the risk of a chronic disease increases dramatically if elite athletes live a sedentary lifestyle after quitting sports. 

Regularly performed training induces a variety of metabolic, functional and morphological adaptations in athletes. Football players have to develop strength, agility, endurance and speed features in order to meet the demands of this multifactorial game. As a result of combined training, football players develop versatile adaptations in neuromuscular, respiratory and cardiovascular systems [39]. For example, Anderson et al. [40] reported that football players have larger muscle fibers than sedentary individuals. Further, high-intensity training is associated with higher bone mineral density, composition and enhanced bone geometric properties [41]. Some studies revealed that the chronic accumulation of exercise stress produces adaptations, such as developed morphological, functional and electrical characteristics of the heart in athletes [42,43]. Depending on the type of exercise performed, different adaptations occur in athletes’ various physiological systems. In order to protect the molecular/cellular/biochemical changes that the athletes develop throughout their active sports career, it was reported that they should continue to exercise [34,38,44]. 

According to some studies [45,46,47,48], former athletes mostly maintain a physically active lifestyle in late adulthood. However, a considerable amount of athletes (approximately 25%) decline their physical activity level or live sedentary lifestyles after retirement from competitive sports [49,50]. Some studies [44,51] reported that athletes are unable to participate in physical activities because of previous injury or pain even if they would like to continue to participate in a high level of sport activities. Without the sense of competition, former athletes may not prefer to participate in physical activities [44]. Furthermore, former athletes may have additional psychological barriers preventing them from participating in physical activities. In their study, Tracey and Elcombe [21] addressed that a strong athletic identity may discourage former athletes from engaging in exercises. They also noted that chronic and acute injuries may contribute to inactivity and psychological consequences of injury may deter physical activity behaviors. Furthermore, the experience of struggling to remain fit or maintain sports performance may cause former athletes to feel embarrassed to participate in exercises [21,51]. Besides, socioeconomic or sociocultural differences may affect the participation in physical activities [52,53]. As a result of decreased participation in physical activities, the biological aging process of former football players (involving both body composition and cardiorespiratory functions) may be influenced negatively. 

The aim of this study was to compare some health-related parameters, such as serum lipids, body composition, cardiorespiratory functions, blood counts and thyroid functions, of former football players according to their physical activity habits after retirement. Our study results revealed that sedentary former football players show a higher cardiovascular risk profile: they had higher body weight, LDLc, BF% and BMI values. It is well known that body composition, serum lipids concentrations and body fat percentages are important anthropometric predictors of coronary heart disease, type 2 diabetes and metabolic abnormalities [54,55,56]. Previous studies [56,57,58] reported that high body mass index, low-density lipoprotein cholesterol and body fat percentage were associated with a higher metabolic risk profile. It was reported that obesity and overweight is one of the leading risk factors for mortality [56]. Moreover, Bianchini et al. [59] revealed that adiposity can cause some cancers, such as colon, breast, kidney and endometrium cancers.

Hyperphagia and inactivity are two major causes of obesity [60]. Thus, weight control can be achieved by decreasing energy intake and increasing physical activities. Moreover, obesity is sometimes linked to endocrine abnormalities, including thyroid dysfunction. Thyroid dysfunction is associated with weight gain and metabolic rate [61]. The hormones of the thyroid gland, FT_3_ and FT_4_, are known to play a role in metabolic rate and weight gain [62]. In our study, thyroid functions were found to be normal in both groups. However, we have found that the body weight and body fat percentage of sedentary former football players is significantly higher (approximately 10 kg) compared to physically active former football players. A number of studies [63,64] showed that trained athletes have favorable serum lipids and body profiles. However, some studies [65,66] reported that former athletes who dramatically reduced their physical activity showed a remarkable increase in BMI, body weight and body fat percentage. Arliani et al. [67] evaluated the health of former Brazilian Professional football players and reported that former footballers gained extra weight, increased body mass index and body fat percentage after ceasing regular training and not maintaining an active lifestyle. In a study on elite runners by Marti et al. [65], they reported a body weight increase and inner fattening in those former runners who decreased their level of physical activity. The study of Pihl et al. [66], which compares sedentary former football players and those who are physically active, confirmed that physical activity acts as a powerful predictor of weight management. 

A decrease in the level of physical activities of those who stopped their active sports career leads to a body weight increase and some negative changes in their serum lipids [68,69]. Some studies also stated that serum lipids are negatively affected by weight gain and this subsequently increases the risk of cardiovascular diseases [70,71]. Although no statistical differences were found in our study, Tg, Tc and LDL values were found to be higher in SG than AG. As to the relationship between serum lipids and cardiovascular diseases, our findings are consistent with the results of other studies [72,73,74] and the risk of cardiovascular diseases is indirectly less in former football players who have a physically active lifestyle. Similarly, Zaccagni et al. [69] reported that the habit of regular physical activities positively affects some biological aging processes, such as body composition and cardiorespiratory functions, in former elite athletes. Lynch et al. [75] also reported that former athletes who have a physically active life have a better body composition and less cardiovascular disease risks than sedentary ones. Vingard et al. [76] reported that old athletes who walk 2–3 times a week for more than 30 min and regularly exercise are healthier than those who do not do regular exercise. 

In the research of Sanchis Gomar et al. [77] that studied the effects of high-intensity endurance training, they reported that the left atrial diameter of the former endurance athletes is wider than the general population. Although it is known that there is a relationship between the left atrial diameter/volume increase and an increase in atrial fibrillation risk, no atrial fibrillation was diagnosed in old aged athletes who took part in their research. Furthermore, they have reported that further research is needed in order to enlighten the clinical results connected with morphological changes in veteran athletes [77].

Although it is well documented that elite athletes live longer and are healthier than the general population [10,69,78], some chronic adaptations of high-intensity training and their effects on health are still being studied. It is thought that training regularly improves the cardiovascular system. As a chronic effect of training, the heart and vascular structure widen, especially in endurance athletes [79]. Green et al. [80] reported that athletes’ arteries have a wider lumen diameter and thinner walls. Furthermore, Sanchis Gomar et al. [77] investigated the long-term effects of high-intensity endurance training on left atrial volume in former athletes and reported larger left atrial dimensions in former highly trained athletes compared with their younger peers. Pellicia et al. [81] carried out a study on elite athletes and they diagnosed no cardiovascular problems, no deterioration in global LV systolic function, no changes in LV systolic function and no wall motion abnormalities for 17 years after they gave up training. However, they reported that these former athletes’ left atrial dimension and left ventricular cavity are bigger but this cardiac remodeling causes no health problems. Furthermore, as the athletes get older, no interpretations are made about the clinical effects of the expansion of the left atrium over a longer period of time [81]. 

As a result of ECG assessments in our research, 12 former athletes who took part in high-level football training were diagnosed to have intraventricular conduction delay and widening of the QRS complex and were diagnosed to have anterior fascicular block. It was reported that elite athletes have a slower heartbeat, increased parasympathetic responses for structural cardiac adaptations and non-homogenic repolarization of ventricles. As a result of the mentioned chronic adaptations, rhythm and conduction differences, morphological changes in QRS complex and repolarization anomalies may occur [82,83]. Besides, Macchi et al. [84] reported that these cardiac adaptations related to training do not completely return to normal after giving up training. Although ECG anomalies are considered to be normal in athletes, a study conducted by Pellicia et al. [85] investigated ECG changes of 12,550 athletes over a 1–17 year period and reported that the abnormal ECG findings in healthy looking young athletes may represent the initial expression of underlying cardiomyopathies. Therefore, it is important to clinically follow athletes who have such ECG patterns. Of all 12 intraventricular conduction delays detected in our study, 9 were in the sedentary group and 3 were in the physically active group. The fact that we see intraventricular conduction delay 3 times more in the sedentary group suggests that continuing physical activities after quitting sports may have an effect on ECG patterns. However, more detailed research is needed on this topic. 

In our study, another health indicator analyzed was the respiratory system. It is known that the respiration volume and capacity decreases after the age of 25 years [86,87]. The changes in the elasticity of lungs, weakening of respiration muscles and disorders in gas changes together with aging particularly cause FVC and oxygen uptake to decrease [87,88,89]. Although athletes have an advanced respiratory system as a result of training compared to inactive individuals, the volume and capacity of lungs decreases in athletes in a similar manner when they age. McClaran et al. [90] reported that even in over 60-year-old individuals who train aerobically and who have almost twice more maximal oxygen uptake than expected depending on age, FEV1 and MVV values decrease by approximately 12% in 6 years [90]. The lifestyle after giving up sports is thought to affect the respiratory system. For example, in Eker et al.’s research [91], it was reported that the expected FEV1 values of former football players who do not exercise regularly were statistically significantly lower (p < 0.05) than an age matched group of footballers. In our study, former athletes who were more physically active showed significantly higher FEV1 values, both as an absolute value and as predicted values according to the age-matched norms of European Respiratory Society, compared to inactive former football players.

Retirement from sports career can be a great struggle for football players. In a review, it was reported that former athletes may experience psychological and physiological problems, such as acute depression, identity confusion, alcohol and substance abuse and eating disorders. However, there are insufficient studies that deal with health problems among former football players. 

The limitations of this study include a relatively modest sample size, lack of blinding and lack of gender comparison. As another limitation, diet as a potentially important determinant of weight gain and serum lipid levels was not assessed. Randomized longitudinal studies would be much more powerful to clarify the interactive effects of lifestyle, diet and health parameters. Despite these limitations, we believe that our results are valuable for encouraging the participation of physical activities after retirement from football. 

## 5. Conclusions

In conclusion, when the parameters of blood counts and thyroid functions are evaluated considering reference ranges, the values of both groups are seen to be within the normal ranges. In the light of ECG results, neither group was seen to have a pathology. However, 12 former athletes were found to have intraventricular conduction delay. The body weight and serum lipids of former football players who engage in a physically active lifestyle result in a decreased cardiovascular disease risk in this group compared to former players who adopt relatively sedentary lifestyles. The results of this study suggest that former elite athletes should maintain physically active lifestyles in accordance with recommendations from the American College of Sports Medicine [92] in order to maintain their health and reduce the risk of disease and disability in the later years of life.

## Figures and Tables

**Table 1:** Physical Variables.

Variables	AG (n = 30)	SG (n = 30)	p	ES
Age (year)	43.4 ± 4.1 (41.1; 45.2)	43.0 ± 4.4 (40.8; 45.4)	0.715	0.09
Height (cm)	177.7 ± 5.5 (175.6; 179.8)	176.3 ± 4.6 (174.6; 178.0)	0.301	0.28
Weight (kg)	79.5 ± 9.3 (76.0; 83.0)	88.5 ±11.6 (84.2; 92.8)	0.002	0.85
BF% (%)	19.3 ± 3.3 (18.1; 20.6)	24.5 ± 4.6 (22.8; 26.2)	0.000	1.30
BMI (kg/m^2^)	25.0 ± 2.3 (24.3; 26.0)	28.4 ± 3.0 (27.3; 34.90)	0.000	1.27

Values are means ± SD (95% Confidence Interval); AG = physically active group; SG = sedentary group; BF% = body fat percentage; BMI = body mass index; ES = effect size.

**Table 2:** Spirometry Parameters.

Variables	AG (n = 30)	SG (n = 30)	p	ES
FVC (L)	4.97 ± 0.67 (4.72; 5.22)	4.74 ± 0.49 (4.56; 4.93)	0.132	0.39
FVCp (%)	103.50 ± 11.35 (99.26; 107.74)	98.77 ± 10.27 (94.93; 102.60)	0.096	0.44
FEV1 (L)	4.08 ± 0.51 (3.89; 4.27)	3.78 ± 0.49 (3.60; 3.96)	0.025	0.60
FEV1p (%)	103.70 ± 9.24 (100.25; 103.74)	96.60 ± 12.19 (92.05; 101.15)	0.014	0.66
FEV1/FVC % (%)	82.21 ± 4.78 (80.43; 84.44)	79.98 ± 8.69 (76.73; 83.22)	0.408	0.32
PEF (L/s)	8.96 ± 1.80 (8.29; 9.64)	8.44 ± 2.17 (7.63; 9.25)	0.310	0.26
FEF 25–75 % (L/s)	4.26 ± 1.01 (3.88; 4.63)	3.92 ± 1.17 (4.48; 4.35)	0.234	0.31
MVV (L/min)	140.79 ± 18.81 (133.76; 147.81)	137.60 ± 23.36 (128.22;146.32)	0.563	0.15

Values are means ± SD (95% Confidence Interval); AG = physically active group; SG = sedentary group; FVC = forced vital capacity; FEV1 = forced expiratory volume in the first; MVV = maximal voluntary ventilation; PEF = peak expiratory flow; FEF = forced expiratory flow at 25–75%; and ES = effect size.

**Table 3:** Blood Parameters.

Blood Parameters	Variables	AG (n = 30)	SG (n = 30)	RR	P	ES
Serum Lipids	Tg (mg/dL)	153.18 ± 91.81 (118.9; 187.5)	191.54 ± 126.14 (144.4; 238.7)	0–150	0.162	0.35
	Tc (mg/dL)	205.63 ± 38.15 (191.4; 219.9)	223.03 ± 35.71 (209.7; 236.4)	0–200	0.073	0.47
	LDLc (mg/dL)	118.11 ± 28.23 (107.6; 128.7)	130.91 ± 30.41 (119.6; 142.3)	0–100	0.046	0.44
	HDLc (mg/dL)	45.08 ± 10.73 (41.1; 49.1)	43.43 ± 9.41 (39.9; 46.9)	40–60	0.668	0.16
Blood Counts	RBC (×10^6^/μL)	5.04 ± 0.26 (4.9; 5.1)	5.24 ± 0.57 (5.0; 5.5)	4–6	0.075	0.45
	WBC (×10^3^/μL)	7.01 ± 1.86 (6.3; 7.7)	6.67 ± 1.38 (6.15; 7.18)	4.8–10	0.615	0.21
	Hct (%)	43.79 ± 2.49 (42.9; 44.7)	43.69 ± 2.59 (42.7; 44.7)	35–52	0.344	0.04
	Hb (g/dL)	14.92 ± 1.08 (14.5; 15.3)	14.77 ± 1.07 (14.4; 15.2)	12–16	0.225	0.14
Thyroid Functions	TSH	1.80 ± 0.80 (1.5; 2.1)	0.86 ± 0.88 (1.5; 2.2)	0.5–4.7	0.988	1.12
	FT_3_	3.27 ± 0.37 (3.1; 3.4)	3.44 ± 0.25 (3.4; 3.5)	2.3–4.2	0.040	0.54
	FT_4_	1.27 ± 0.15 (1.2; 1.3)	1.28 ± 0.15 (1.2; 1.3)	0.9–2.0	0.694	0.07

Values are means ± SD (95% Confidence Interval); AG = physically active group; SG = sedentary group; RR = reference range; Tg = triglyceride; Tc = total cholesterol; LDLc = low density lipoprotein cholesterol; HDLc = high density lipoprotein cholesterol; RBC = red blood cell count; WBC = white blood cell count; Hct = hematocrit; Hb = hemoglobin; TSH = thyroid stimulating hormone; FT_3_ = free triiodothyronine; FT_4_ = free thyroxine; and ES = effect size.
